# Effects of Different Intensities of Repetitive Peripheral Magnetic Stimulation on Spinal Reciprocal Inhibition in Healthy Persons

**DOI:** 10.14789/jmj.JMJ23-0039-OA

**Published:** 2024-06-15

**Authors:** WANHONG ZHANG, TOMOFUMI YAMAGUCHI, TOSHIYUKI FUJIWARA

**Affiliations:** 1 Department of Rehabilitation Medicine, Juntendo University Graduate School of Medicine, Tokyo, Japan; 1 Department of Rehabilitation Medicine, Juntendo University Graduate School of Medicine, Tokyo, Japan; 2 Department of Physical Therapy, Juntendo University Faculty of Health Science, Tokyo, Japan; 2 Department of Physical Therapy, Juntendo University Faculty of Health Science, Tokyo, Japan

**Keywords:** repetitive peripheral magnetic stimulation, reciprocal Inhibition, spinal circuits, h-reflex

## Abstract

**Objectives:**

This study aimed to assess the effect of the spinal circuit of repetitive magnetic stimulation (rPMS) on the soleus muscle among healthy subjects.

**Methods:**

Nineteen healthy adults were included in this study. Intermittent rPMS was applied to the left soleus muscle for 20 minutes. We applied different intensity rPMS (high-intensity, low-intensity, and non-stimulation) in different three days. RI (reciprocal inhibition) from the tibialis anterior to the soleus muscle with an inter-stimulus interval (ISI) of 2ms and 20ms was assessed before, immediately after and 30 minutes at each session.

**Results:**

Two factor repeated measure ANOVA test showed a significant interaction (F_2,33_ = 9.688, p < 0.001) between tasks and time in the RI ratio 2ms. Post-hoc analysis showed that RI ratio 2ms significantly differed from those immediately after, and 30 min after high-intensity rPMS (p = 0.001 and p = 0.003, respectively). A significant difference was observed between high-intensity rPMS and non-stimulation immediately after the stimulation (p = 0.003). However, no significant difference was found in the RI ratio 20ms between all the intensities (p > 0.05).

**Conclusion:**

This study demonstrates that high-intensity rPMS can effectively modulate spinal circuits, as evidenced by the decreased RI in healthy individuals. This suggests the potential use of rPMS as a therapeutic intervention for patients with muscle weakness. Disinhibition of the RI may lead to a more effective contraction of the target muscle. This effect could be expected to strengthen the muscles and alleviate paralysis, making it a promising avenue for future research and clinical applications in the field of rehabilitation. Further investigation is warranted to explore the precise mechanisms underlying the observed effects and to optimize the parameters of rPMS for specific clinical populations.

## Introduction

Repetitive peripheral magnetic stimulation (rPMS) and peripheral nerve electrical stimulation are known to induce muscle contraction. Notably, magnetic stimulation is considered less painful than electrical stimulation, as the induced electrical current can directly target deep tissues without penetrating the skin^1)^. This characteristic makes rPMS particularly well-suited for application in patients with chronic pain and muscle weakness. The effectiveness of rPMS extends beyond its application in healthy subjects, where it enhances motor performance^2)^, to benefiting patients experiencing central hemiparesis^3)^. Previous research has also highlighted the therapeutic potential of rPMS in addressing musculoskeletal pain^4, 5)^ and reducing lumbar radiculopathy pain^6)^.

In a comprehensive analysis conducted in 2022, Pan et al. analyzed eight randomized controlled trials (RCTs) involving 170 patients with stroke or other neurological disorders, revealing that rPMS can effectively reduce spasticity in both upper and lower limbs^7)^. Despite these positive findings, the impact of rPMS on spinal circuit remains unclear. While existing evidence suggests the potential of rPMS as a valuable treatment for promoting motor performance, further exploration is needed to elucidate its effects on spinal circuitry.

Reciprocal inhibition (RI) between agonist and antagonist muscle is mediated by the Ia inhibitory interneurons^8^^-^^10)^, which are responsible for the achievement of smooth movement between these muscles^10, 11)^. RI plays a crucial role in modifying aspects of locomotor and other functional abnormalities associated with conditions such as stroke, spinal cord injuries, and other chronic disorders of motor control, ultimately contributing to more effective function^12)^. For instance, spinal cord injuries in humans are characterized by heightened stretch reflexes and flexor afferent reflexes, coupled with a reduction in RI. These anomalies are believed to contribute to spasticity^13)^. Conditioning the RI pathway holds potential for enhancing spinal cord function in individuals with incomplete spinal cord injuries or other neurological disorders.

Therefore, our study aims to investigate the effects of repetitive peripheral magnetic stimulation (rPMS) on spinal RI using the H-reflex. This exploration seeks to advance our understanding of how rPMS may modulate spinal circuitry and potentially offer therapeutic benefits for individuals with incomplete spinal cord injuries or other neurological disorders.

## Materials and Methods

### Participants

19 healthy volunteers (8 females and 11 males, with a mean age of 27.79 ± 5.27 years) participated in this single-blind crossover study. All of them met the following inclusion criteria: (1) age between 20-50 years; (2) no history of orthopedic surgery in the lower limb; (3) no medical history of nervous system disease (including epilepsy); (4) not using drugs affecting the central nervous system; (5) without pacemaker or other metallic orthopedic implants in the body. All subjects provided written informed consent before participating in the study. The experiment commenced on July 5, 2022, and concluded on August 30, 2023.

### Repetitive peripheral magnetic stimulation (rPMS)

**Figure 1:**
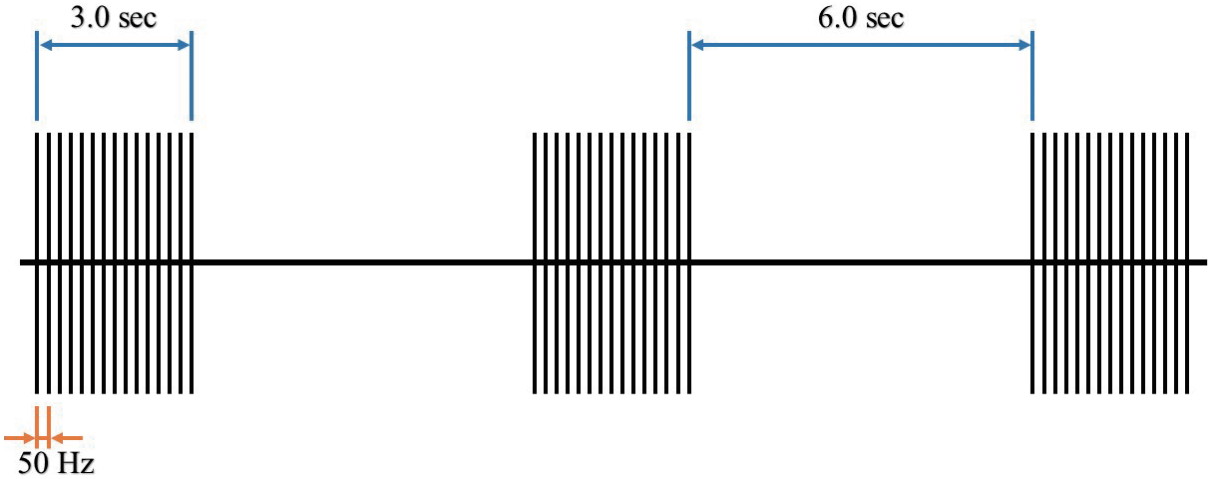
Illustration of the stimulation frequency. The stimulation frequency is set at 50Hz, and each train consists of 10 pulses delivered 3 seconds, followed by a 6-second interval, the stimulation will last for 20 mins.

RPMS was delivered using a coil connected to a Salus Talent Pro (REMED, Korea). Subjects were instructed to sit in a relaxed position, and the stimulation coil was positioned on the calf of the left limb, targeting the top of the Achilles tendon to stimulate the soleus muscle belly. Stimulation parameters were a frequency of 50Hz, a 3-second stimulation (10 biphasic pulses, each pulse width 0.02 seconds) with a 6-second interval, and a total stimulation duration of 20 minutes (Figure 1). Notable side effects, such as pain and a burning sensation were carefully managed by adjusting the stimulation intensity according to the subject’s pain threshold. Electrodes were removed as a precautionary measure to prevent potential skin burns caused by the heat generated during stimulation.

### H-reflex

The H-reflex was elicited by stimulating the tibial nerve at the popliteal fossa using 1ms rectangular pulse. Conditioning stimulation to the common peroneal nerve (CPN) was delivered below the fibular head, with a stimulus intensity of 1.0×motor threshold (MT). MT was defined as a 100μV response of the tibialis anterior (TA). The reflexes were recorded by disc electrodes placed over the soleus muscle. The sensitivity of the H-reflex to facilitatory and inhibitory conditioning effects has been shown to depend crucially on its size^14)^. Hence, when measuring the effects of conditioning stimuli, the size of the test soleus H-reflex (testH) amplitude maintained at 20-25% of the maximum M amplitude (Mmax) for each block of trials. The amplitude of Mmax before and 30min after the stimulation was measured to ensure there was no displacement of stimulating electrodes during movement.

### Reciprocal inhibition (RI)

RI was assessed using a soleus H-reflex conditioning-test paradigm^15)^. Ten conditioned and ten test H-reflexes were measured, and the mean value of the ten measures was calculated. The amount of RI was defined as mean conditioned H- reflex amplitude divided by mean test H-reflex amplitude. To confirm optimal disynaptic RI, we checked the H-reflex at a conditioning-test inter- stimulus interval (ISI) of 0, 1, and 2ms at the beginning of each session. The ISI were set at 2ms and 20ms to trigger inhibition through separate mechanisms^16)^. Inhibition at an ISI of 2ms is called disynaptic RI (RI2ms) and is mediated by a spinal glycinergic disynaptic inhibitory pathway^17, 18)^. Inhibition at an ISI of 20ms (RI20ms) is called short-latency presynaptic inhibition, which is thought to result from presynaptic Ia inhibition of afferent fibers that mediate the H-reflex^16)^. We assessed RI before, immediately after, and 30 minutes after each stimulation.

### Experimental Procedure

**Figure 2:**
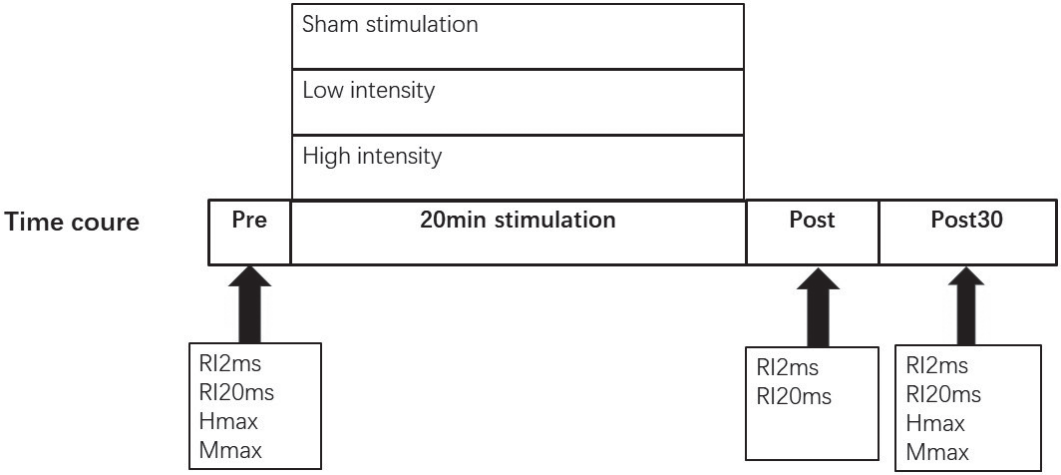
Experimental procedure. Nineteen subjects participated in three sessions: (1) non-stimulation; (2) low-intensity; (3) high-intensity. All three sessions are delivered in 20 minutes. Measurements of reciprocal inhibition at ISI 2ms and 20ms were taken before, immediately after(post), and 30 minutes after (post30) each stimulation session.

Three types of rPMS (high-intensity stimulation, low-intensity stimulation and non-stimulation) were randomly applied on separate days. High- intensity was defined as the maximal intensity the participants can tolerate without pain and induce muscle contraction. The low-intensity is the motor threshold of soleus muscle, with intensity stimulation evoking minimum muscle twitch. For the non-stimulation, coil was placed in the same position as high and low-intensity stimulation, but stimulus intensity was set at 0. We assessed RI before, immediately after rPMS (post) and 30 minutes after rPMS (post 30) (Figure 2).

### Statistical analyses

The Kolmogorov-Smirnov test was utilized to assess the normal distribution of all data, and no significant deviation from normality was observed. Two-factor repeated measures ANOVA was used to analyze the effects of types of rPMS (high-intensity stimulation, low-intensity stimulation, and non- stimulation) and time points (before, immediately after and post30) of the differences in RI ratio. The changes of testH and Mmax amplitudes of each stimulation were analyzed with one-factor repeated measure ANOVA with main factor of time.

For post hoc comparisons, Scheffe’s test for multiple comparisons was employed to analyze the results of all data. Results with P values < 0.05 were considered statistically for all analyses. Statistical analyses were conducted using SPSS 29.0 (IBM Corp, Armonk, NY, USA) for Windows.

## Results

The mean testH amplitude was 3.54 ± 1.49 mV before high-intensity stimulation, 3.19 ± 1.55 mV before low-intensity stimulation, 3.70 ± 1.63 mV before non-stimulation. There were no significant differences in the baseline of the testH amplitudes among different paradigms (F = 0.510, p = 0.603). One-way repeated measure ANOVA showed no significant main effect of time (before, immediately after, 30min after) on the testH amplitudes in high- intensity stimulation (F = 0.053, p = 0.948), low- intensity stimulation (F = 0.154, p = 0.858), and non- stimulation (F = 0.063, p = 0.939).

The mean Mmax amplitude was 18.25 ± 4.76 mV before high-intensity stimulation, 18.41 ± 4.52 mV before low-intensity stimulation, 18.83 ± 5.00 mV before non-stimulation. There were no significant differences in the baseline of the Mmax amplitudes among different paradigms ((F = 0.185, p = 0.981). One-way repeated measure ANOVA showed no significant main effect of time (before and 30min after) on the Mmax amplitudes in high-intensity stimulation (F = 3.888， p = 0.058), low-intensity stimulation (F = 0.528, p = 0.473), and non-stimulation (F = 0.819, p = 0.373).

**Figure 3:**
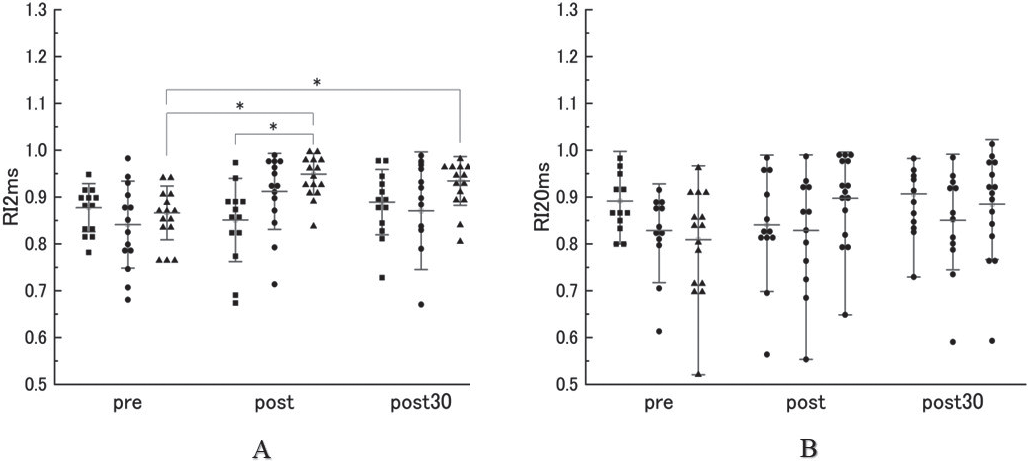
Changes in reciprocal inhibition (RI) with an ISI of 2ms (RI ratio 2ms) and 20ms (RI ratio 20ms) under different stimulation intensities. Asterisks indicate significant differences. Filled square represents non-stimulation intensity; filled circle represents low-intensity; filled triangle represents high-intensity. Significant difference was observed between pre RI ratio 2ms and post RI ratio 2ms (p = 0.001) as well as pre RI ratio2ms and post 30 RI ratio 2ms (p = 0.003) following the high-intensity rPMS intervention. There is also a significant difference between the non-stimulation post RI ratio 2ms and high-intensity post RI ratio 2ms (p = 0.003) (Figure 3A). However, no significant difference was found in the RI ratio 20ms between all the intensities (Figure 3B).

Furthermore, at baseline, the amount of RI ratio did not exhibit a significant difference between different stimulation types. However the analysis (ANOVA) revealed a significant interaction (F_2,33_ = 9.688， p < 0.001) between tasks and time in the RI ratio at 2ms. Post-hoc testing indicated that high- intensity rPMS significantly increased RI ratio 2ms post and post30 compared to pre (p = 0.001 and p = 0.003, respectively). Additionally, a significant difference of RI ratio 2ms was observed between the value of high-intensity and non-stimulation rPMS immediately after the stimulation (p = 0.003) (Figure 3A).

The RI ratio is calculated by dividing conditioned H-reflex amplitude by test H-reflex amplitude, an increase in the RI ratio represents a decrease in inhibition, while a decrease in the RI ratio represents an increase in inhibition. We also found no significant difference in the RI ratio 20ms between all the intensities (p > 0.05) (Figure 3B).

## Discussion

In this experiment, we applied 3 intensities of rPMS to the soleus muscle and utilized RI to access the effect of the spinal circuit. We observed that high-intensity rPMS led to disinhibition with an ISI of 2ms. RPMS on the soleus muscle induced a visible, strong plantar flexion of the ankle, forcing the soleus muscle to contract, similar to voluntary plantar flexion. Higher intensity rPMS can activate more muscle spindles; the activated Ia afferent neuron can fire more alpha motor units which can generate stronger contraction of the soleus muscle. The contraction of the soleus muscle generates stronger inhibition to the tibial anterior (TA), and weaker activity of the TA can also explain the disinhibition effects of RI from TA to soleus found under high-intensity stimulation. And we also considered the reason why only RI2ms is disinhibited. In this experiment, all subjects are healthy people with normal RI, making interference of RI20ms more challenging than in patients with impaired RI. The simplest explanation for this result is that modulation of the interneurons mediating short-latency presynaptic inhibitions might be weak compared to the effects on the circuit responsible for disynaptic RI. Applying high-intensity rPMS to patients with neurological disease or paralysis, who may struggle with voluntary contraction to control the target muscle, could potentially induce disinhibition of the RI. This could also result in more effective contraction to the target muscle, offering the potential to strengthen muscle and alleviate paralysis, making it a promising avenue for future research and clinical applications in the field of rehabilitation. The non-invasive nature of rPMS, along with its capacity to induce plasticity in spinal circuits, highlights its potential for future research and clinical applications in neuromodulation. Further investigations are needed to elucidate the underlying mechanisms and optimize rPMS parameters for specific clinical populations.

## Funding

This study was funded by the Department of Rehabilitation Medicine, Juntendo University Graduate School of Medicine.

## Author contributions

All authors read and approved the final manuscript.

## Conflicts of interest statement

The authors declare that there are no conflicts of interest.
